# A Treatise on Dislocations and Fractures of the Joints

**Published:** 1851-08

**Authors:** 


Art. VII.—*A Treatise on Dislocations and Fractures of the Joints.* By Sir Astley Cooper, Bart., F. R. S. A new edition, much enlarged. Edited by Bransby B. Cooper, F.R.S., Surgeon to Guy's Hospital. With additional observations, and a memoir of the author. A new American edition. Philadelphia: Blanchard & Lea. 1851.

It is questionable whether any treatise on a surgical speciality ever conferred the same amount of benefit on mankind, that has been derived from this. The original was a work of sterling merit, but each successive edition was an improvement upon its predecessor, for in addition to the new contributions from Sir Astley's constantly enlarging experience, the intelligent practitioners, he sent out prepared for practice, greatly enlarged his field of information by the results of their practice. Even up to the time of his death, Sir Astley was engaged in perfecting his work, and his papers were left with his nephew, Bransby B. Cooper, to be incorporated in a new edition, and the American edition, before us, is based upon the most recent and best of the English editions, and contains some valuable contributions from Professor John C. Warren.

The surgery of dislocations and fractures of the joints can never be too thoroughly studied. In this country, the general practitioner is expected to be ready, at all times, to take hold of these cases, and to treat them properly. Their correct management, requires a great variety of knowledge, which must be at immediate command. A single error may endanger the life of the patient, and even if the major evil may not occur, there may be a deformity that stands as a monument of bad surgery, and a crippled gait that unfits the patient for the business of life. These evils were more frequent before the publication of Sir Astley's work than since that time, and a careful and thorough mastery of its principles by all practitioners, will tend to diminish the evils of bad surgery in dislocations and fractures of the joints.

It is one of the offices of the medical periodicals to keep alive all the great elements that conduce to the success of the healing art, and it is better to reiterate old and valuable truths, than to adventure upon the sea of conjecture, in the utterance of novelties. The great charm of Sir Astley Cooper's works on surgery, is their eminently practical character. No man ever devoted himself more faithfully than Sir Astley to the improvement of the various departments of surgery, and no one has met with greater success. Neither the incessant calls upon his professional services, nor his severe duties as a public instructor, was sufficient to interfere with his career as an author. His works on hernia, on the female breast, on the testes, and the book before us are monuments of his industry and ardent love of his profession, beyond all that marble or brass could speak.

The American edition of this work, recently published, is not only enriched with numerous additions prepared by Sir Astley, but by contributions from the experience of Dr. J. C. Warren, of Boston. The observations of Dr. Warren are on dislocations of the hip joint, and on fractures of the upper part of the thigh bone. They are very instructive.

The typography of this work is excellent, and the wood engravings are clear and forcible illustrations of portions of the text. We commend the American edition to the favor of the medical profession. B.

